# Complete Genome Sequence of a Novel Porcine Circovirus Type 3 Strain, PCV3/CN/Hubei-618/2016, Isolated from China

**DOI:** 10.1128/genomeA.00100-17

**Published:** 2017-04-13

**Authors:** Shengxian Fan, Xugang Ku, Fangzhou Chen, Ying Wang, Xuexiang Yu, Qigai He

**Affiliations:** a State Key Laboratory of Agricultural Microbiology, College of Veterinary Medicine, Huazhong Agricultural University, Wuhan, China; b Cooperative Innovation Center for Sustainable Pig Production, Huazhong Agricultural University, Wuhan, China

## Abstract

In October 2016, porcine circovirus type 3 (PCV3) was identified as a pathogen agent for pigs in the United States. Here, we report the genome sequence of a Chinese PCV3 strain, PCV3/CN/Hubei-618/2016. This will help us better understand the epidemiology and genetic characteristics of PCV3.

## GENOME ANNOUNCEMENT

Porcine circoviruses are nonenveloped, single-stranded, small circular DNA viruses that belong to the genus *Circovirus* (1). Porcine circovirus type 1 (PCV1) and porcine circovirus type 2 (PCV2) have genome sequences of 1,759 bp and 1,767 to 1,768 bp in length, respectively. PCV1 was nonpathogenic to pigs (2), while PCV2 was a globally important pathogen that caused various syndromes in pigs (3). Recently, porcine circovirus type 3 (PCV3), with a genomic size of 2,000 bp, was identified as the etiologic agent of porcine dermatitis, nephropathy syndrome, and reproductive failure (4) and caused cardiac and multisystemic inflammation (5).

In December 2015, a total of 14 samples (four brain, six lymph node, and four lung) with productive failure were collected from a 400-sow pig farm in Hubei Province, China, and diagnosed at the Animal Disease Diagnostic Center of Huazhong Agricultural University. The common pathogens related to reproductive failure, such as pseudorabies virus, PCV2, and porcine reproductive and respiratory syndrome virus, were ruled out from the samples. In our retrospective study, 12 of 14 samples were identified as strongly positive for PCV3 using our in-house PCR method. Then, the PCV3 strain PCV3/CN/Hubei-618/2016 was isolated and subjected to complete genomic sequencing using our in-house method.

The genome sequence of PCV3/CN/Hubei-618/2016 is 2,000 nucleotides (nt) in length with a single-stranded circular genome. Open reading frames 1 and 2 (ORF1 and ORF2) are the two major open reading frames in PCV3. In PCV3/CN/Hubei-618/2016, ORF1 was located at nt 223 to 1113 in the positive strand that is associated with virus replication initiation. The code “GTC,” instead of the common start code “ATG,” was observed in the ORF1 gene. The ORF2 gene located at nt 1987 to 1343 in the complementary negative strand encoded a major structural protein. Other ORFs could be identified in the PCV3 genome, such as at nt 69 to 602, nt 616 to 1114, nt 1120 to 1470, and nt 1238 to 1657 in the positive strand and nt 1470 to 1120 in the negative strand. The functions of these ORFs are unknown.

The ORF1 gene, ORF2 gene, and whole-genome sequence of PCV3/CN/Hubei-618/2016 share, respectively, 99.7%, 99.2%, 99.3%, 99.8%, 99.2%, and 99.8%; 99.7%, 98.6%, 98.4%, 98.1%, 98.6%, and 98.1%; and 99.6%, 99.2%, 99.1%, 99.2%, 99.2%, and 99.2% nucleotide identities with six PCV3 strains identified in the United States: PCV3/USA/MO2015 (KX778720.1), PCV3/USA/SD2016 (KX966193.1), PCV3/USA/MN2016 (KX898030.1), PCV3/USA/29160 (NC031753.1), PCV3/USA/2164 (KX458235.1), and PCV3/USA/29160 (KT869077.1). Variations of the encoded ORF1 and ORF2 proteins were located at the deduced amino acid positions 44, 122, 187, and 260 and 24, 27, 56, 77, 100, and 150, respectively.

The sequence data of PCV3/CN/Hubei-618/2016 will facilitate future studies on the epidemiology and evolutionary characterization of PCV3. Further investigation of the etiology and clinical significance of PCV3 is necessary.

### Accession number(s).

The genomic sequence of PCV3/CN/Hubei-618/2016 has been deposited at GenBank under the accession number KY354039.
